# Vein of Galen Aneurysmal Malformations: An Ultrasonographic Incidental Finding—A Case Report

**DOI:** 10.1155/2012/824284

**Published:** 2012-11-01

**Authors:** S. Stephan, G. Rodesch, E. Elolf, D. Wiemann, G. Jorch

**Affiliations:** ^1^Department of Pediatrics, Otto-von-Guericke-University Magdeburg, 39120 Magdeburg, Germany; ^2^Service de Neuroradiologie Diagnostique et Thérapeutique, Hôpital Foch, Suresnes, France; ^3^Department of Radiology and Neuroradiology, International Neuroscience Institute, Hannover, Germany

## Abstract

*Background*. The Vein of Galen aneurysmal malformation (VGAM) is a rare congenital, cerebral, arteriovenous deformity. Good cross-discipline cooperation is in demand because of associated complications and high mortality. The recognition of the optimal therapeutic window is useful to allow proper management. *Case Report*. We report on the successful treatment of a 2-week-old, healthy girl with a VGAM, which came across in the context of the newborn ultrasonographic screening. After interdisciplinary discussion, 2 embolizations of the VGAM followed without complications—the first in the age of 6 months and the second at 12 months of life. Before and after the intervention, the patient had an age-appropriate development without neurological deficits. *Conclusion*. The endovascular transarterial embolization is described as the treatment of choice. Time and method of intervention depend on clinical signs of the patient. In our case the patient was asymptomatic. So the arteriovenous abnormality was an incidental finding by ultrasound. Because of the natural history of the disease, and the potential severe neurocognitive consequences at long-term followup if left untreated, it was decided to embolize the lesion. Thanks to embolization with glue, good therapeutical and clinical results could be obtained with normal neurological development.

## 1. Introduction

Vein of Galen aneurysmal malformation (VGAM) was first described by Steinhel in 1895 [1]. It is a rare congenital, cerebral, arteriovenous abnormality with an incidence of 1 : 25000. The VGAM develops during the 6th to 11th weeks of gestation and can already be diagnosed prenatal by ultrasound [2]. But most often the VGAM is detected in the postnatal period [3]. Multiple shunts with brain arteries are the cause of the aneurysmatic enlargement of the big unpaired cerebral vein, also called as *Vena Galeni*. Depending on the age of the patient and on the anatomy and angioarchitecture of the malformation, there are different symptoms. In the neonate (choroidal type of the VGAM—multiple arteriovenous shunts because of a contribution of all choroidal arteries before emptying into a venous pouch), evidence of volume overloading is often dominating: raised cardiac output per minute, tachycardia, cardiomegaly and cardiac insufficiency (high-output-failure), pulmonary hypertension, respiratory distress syndrome, and pulmonary oedema up to multiorgan failure. In infancy (mural type of the VGAM—less, but bigger shunts as direct arteriovenous fistulas within the wall of the median prosencephalic vein of Markowski), hydrocephalus, macrocrania, and developmental retardation are relevant. Rarely seen in VGAM are epileptic seizures, showing the damage of the brain. In different areas of the world, the clinical presentation can differ from the general descriptions in the literature [4]. Left untreated, the mortality of newborns with severe cardiac insufficiency amounts to 100%, in infancy 72% [5]. The safest targeted treatment of a VGAM and also therapy of choice is the endovascular embolization—primarily transarterial using a special kind of glue [6–8]. Higher rates of complications and mortality are described for other therapeutical methods (transvenous embolization, surgical treatment) [9]. Time and method of endovascular embolization depends on clinical signs and symptoms of the patient [3, 10]. The *Bicêtre*-score is helpful to evaluate the therapeutical management of neonates with VGAM (Table 1). It is based on the experience of the research group of Lasjaunias with over 300 patients with VGAM [6]. Using the scoring system, the cardiac, cerebral, respiratory, hepatic, and nephrological state of the patient can be assessed. If cardiovascular and neurologic symptoms are stable (*Bicêtre*-score > 12), it is recommended to postpone treatment up to the age of 5 to 6 month [6, 11, 12]. With a *Bicêtre-*score of 8–12 points, an emergency endovascular embolization should be done. In case of profound neurological defects or medically uncontrollable cardiac insufficiency (*Bicêtre*-score < 8), there is no indication for an invasive procedure [6].

In former days, the introduction of embolization had a bad outcome (high mortality and morbidity) both in neurosurgical and in conservative treated patients [13, 14]. Recent works of different research groups demonstrated higher survival rates, normal growth and at most mild neurological deficits in VGAM-patients who got a timely, therapeutic embolization [6, 11, 15, 16]. 

## 2. Case Report

**Figure 1:**
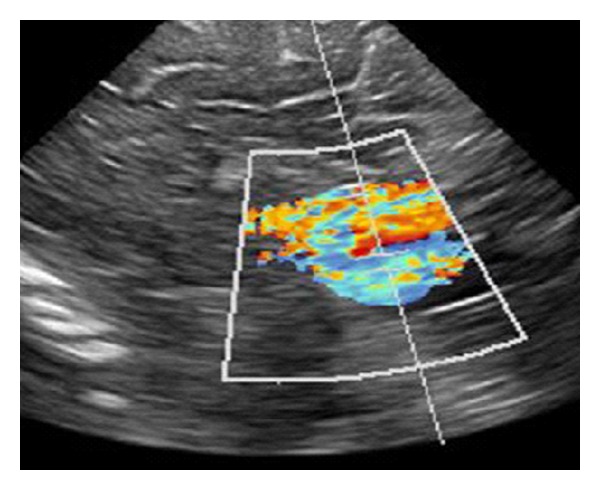
Colour duplex sonography: VGAM with mixed venous and arterial flow profiles.

**Figure 2:**
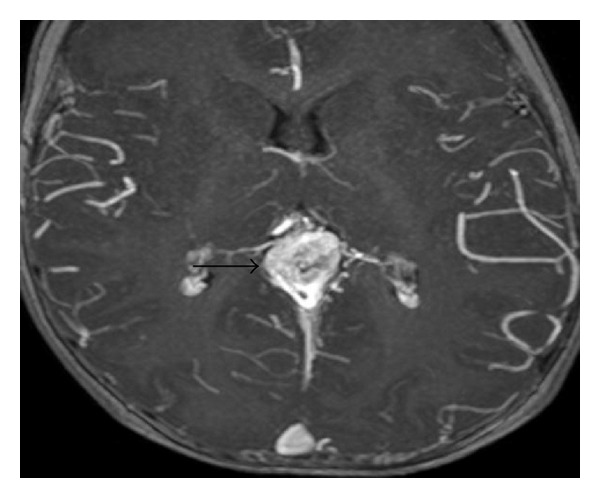
MRI (MPRAGE with contrasting agent): greatly expanded Vein of Galen (arrow).

**Figure 3:**
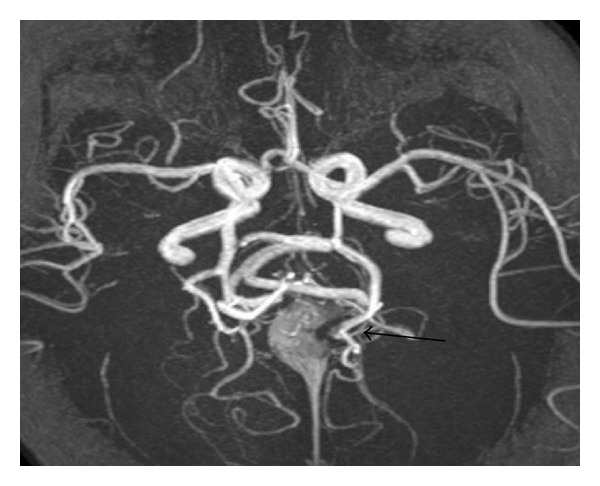
Angio-MRI (TOF): arterialization of the Vein of Galen and consecutive dilatation (arrow).

In a clinically healthy, female, full-term newborn (normal history of gestation and birth), a tumour with suspicion of a pineal cyst was diagnosed in the postpartum ultrasonographic screening. At admission we saw a 2-week-old, age-appropriate developed child with normal internal and neurological status. The ultrasonographic incidental finding, realized by using a colour duplex sonography, a VGAM (maximum diameter 2 cm) with mixed venous and arterial flow profiles, was seen. The remaining cerebral result presented as normal (Figure 1). These findings were confirmed by MRI (Figures 2 and 3). 

**Figure 4:**
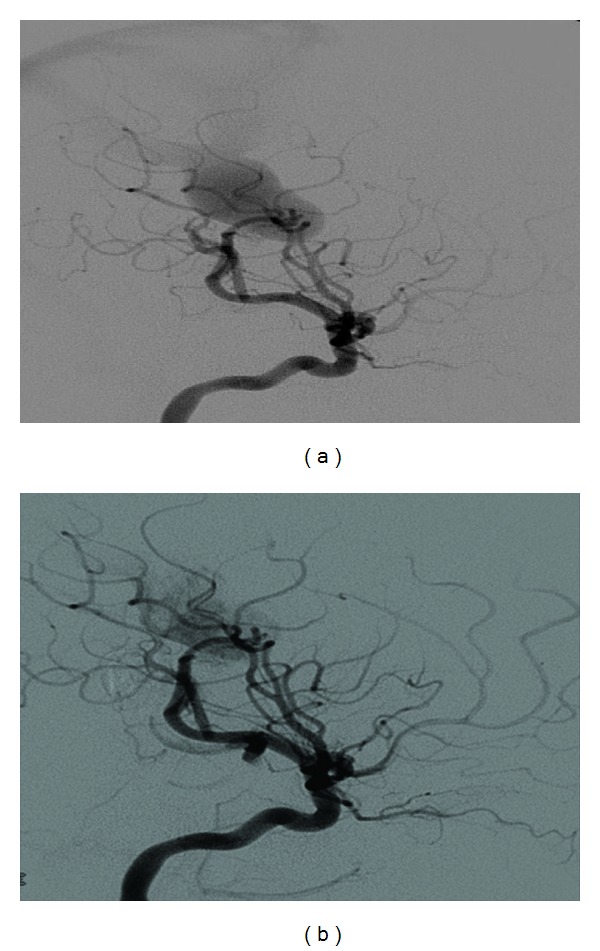
Digital subtraction angiography: internal carotid artery lateral and early filling of the Vein of Galen before (a) and after (b).

**Figure 5:**
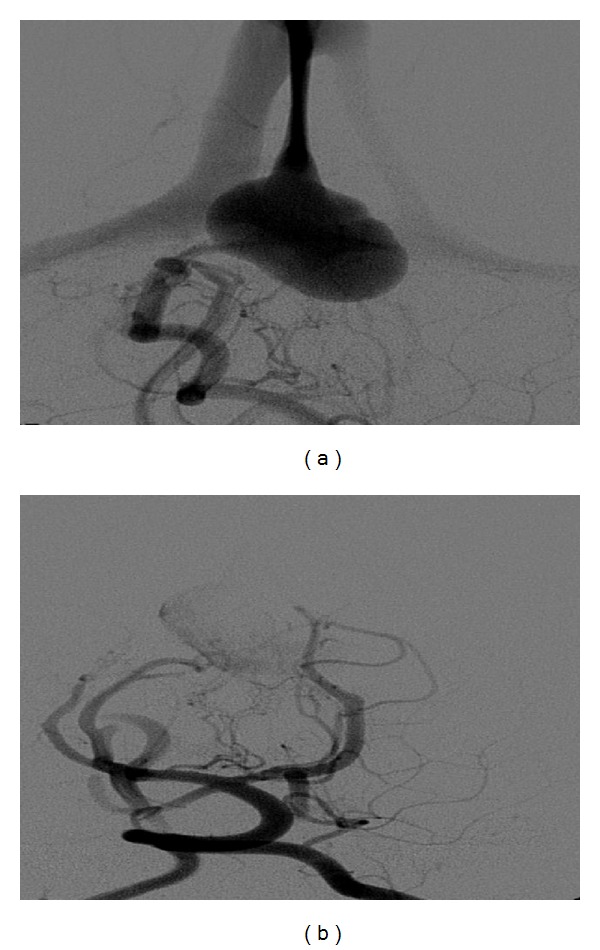
Digital subtraction angiography: vertebral artery ap, with fistula to the Vein of Galen before (a) and after (b).

**Figure 6:**
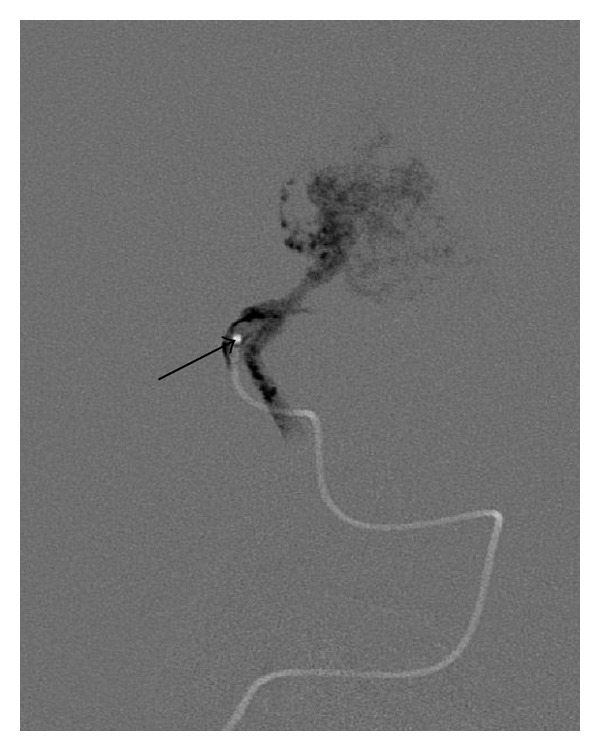
Digital subtraction angiography: Embolisat-Cast (NBCA), (arrow: catheter tip).

Laboratory diagnostics, electrocardiogram, and ultrasound could not find new results or other pathological aspects. After interdisciplinary discussion and calculation of a *Bicêtre*-score >12, a neuroradiological intervention was considered, indicated during the phase of the “optimal therapeutic window.” Thus, at the age of 6 months, a transarterial embolization of 2 afferent vessels was performed using acrylic glue (Figure 6). The dominant blood flow was coming from the right internal carotid artery, fistulous directly to the Vein of Galen. Another fistula to the Vein of Galen was coming from vessels of the vertebral artery (Figures 4 and 5). 

**Figure 7:**
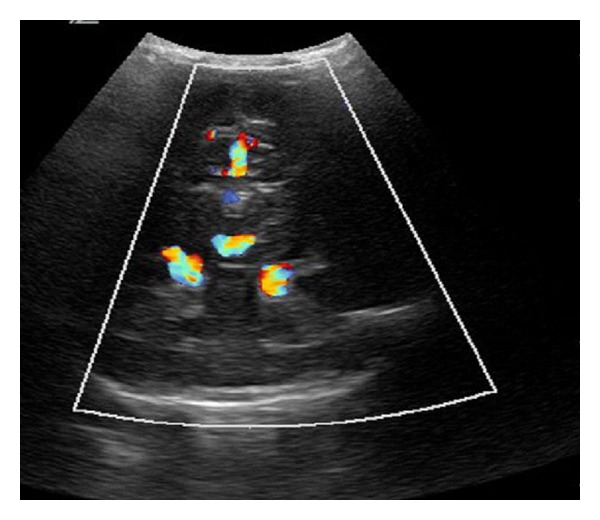
Colour duplex sonography after first embolization: no cystic mass is visible any more.

**Figure 8:**
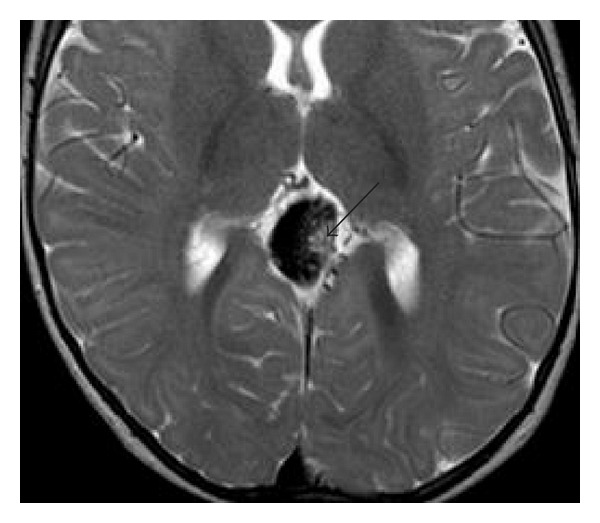
MRI followup control after the first embolization (T2-weighted): dilated Vein Galen after intervention with NBCA-Cast (arrow).

In the follow-up control in the age of 8 months ultrasound and MRI showed a partial closure of the VGAM with normal ventricular system (Figures 7 and 8).

**Figure 9:**
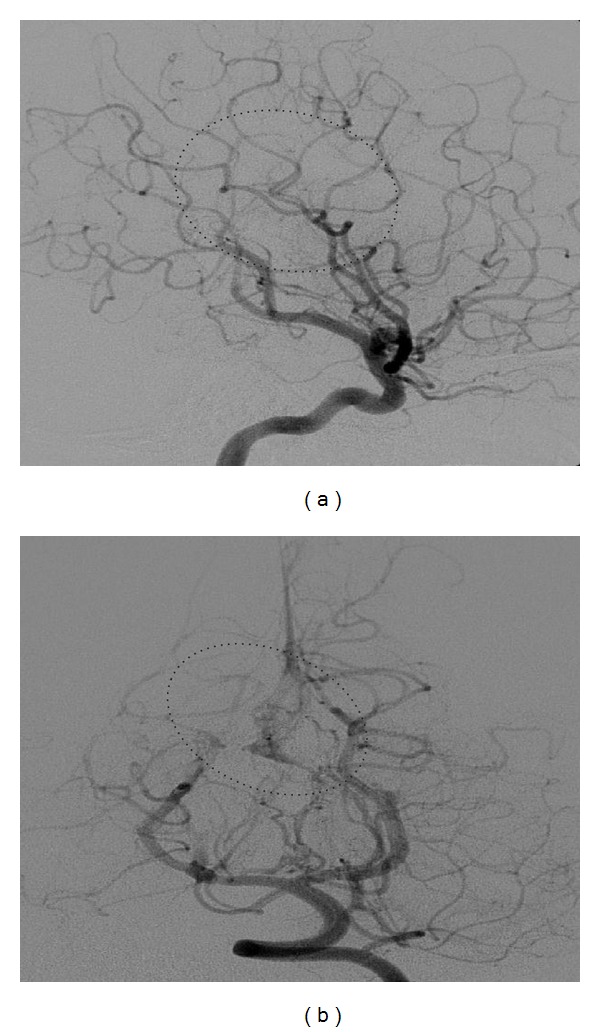
Digital subtraction angiography after second endovascular embolization. (a) Internal carotid artery, a pathological drainage cannot be longer represented (circle). (b) Vertebral artery, small residuum without relevant filling of the Vein of Galen (circle).

After the first procedure, we planned a second embolization. This intervention followed with 12 months of life successfully with the result of a 95-percent closure of the vascular lesion (Figure 9). The remaining microshunt was identified coming from a subependymal artery, which is impenetrable for an embolization catheter (Figure 9(b)). We expect a secondary occlusion of it and no hemodynamic relevant shunts.

**Figure 10:**
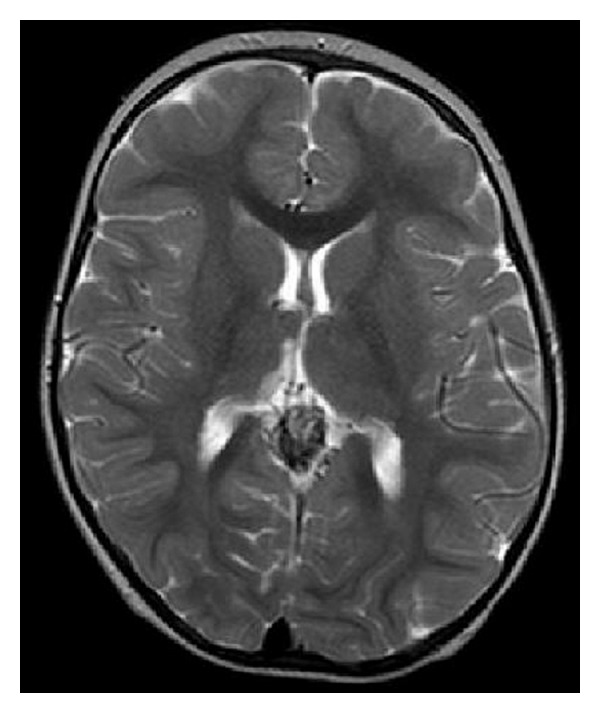
MRI follow-up control after the second embolization (T2-weighted): Vein of Galen still patent, but significant decrease in seizure. NBCA-casts in the sack.

**Figure 11:**
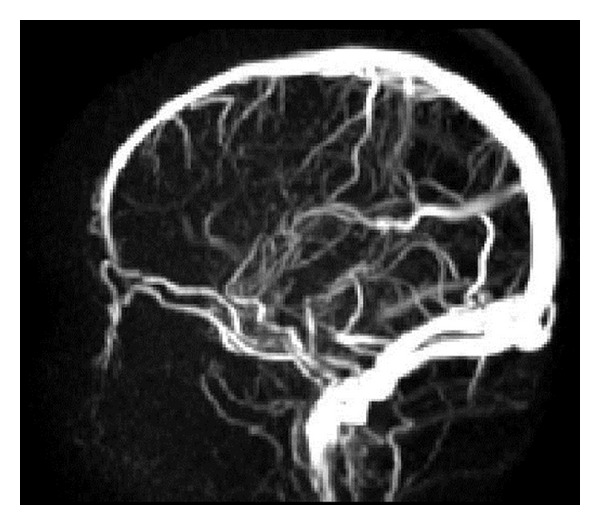
Phase contrast-MRI: no indication of perfusion of the venous aneurysm.

In the following period (4 months later), thrombosis of the malformation with no perfusion of the venous aneurysm and no sign of a venous congestion could be diagnosed by MRI. The venous return was now made by collaterals in the Sinus transversus and Sinus sigmoideus (Figures 10 and 11).

The development—and the neurological status have always been normal before and after the interventions (Table 2).

The next follow-up clinical and radiological inspection of the described patient is provided in 3 years.

## 3. Conclusion

The VGAM can be detected already in the prenatal period [10, 17]. So the diagnosis of an arteriovenous malformation demands an intensive interdisciplinary collaboration of obstetricians, pediatricians, neuroradiologists, and neurosurgeons. If there are already prenatal cardiac insufficiency or/and cerebral damages, the prognosis for the patient is poor [10].

The time and method of therapeutical intervention, respectively, and the endovascular embolization depend on clinical signs and symptoms of the patient [6, 18]. The *Bicêtre*-score is useful for evaluating the therapeutical management of a neonate with VGAM. If cardiovascular and neurologic symptoms are manageable conservatively, the intervention should be done in the age of 5 to 6 months after birth [6, 10].

It has been shown that most of the children who were treated by endovascular embolization showed a normal especially neurological development [6].

Rodesch et al. present about 16 neonates with VGAM and cardiac insufficiency, of which 12 survived effectively after therapy with diuretic medication and embolization and such have shown a normal neurological development. The 4 untreated ones died shortly after birth because of multiorgan failure [18].

Lasjaunias demonstrated with a cohort of 216 patients over a period of 21 years, that 74% of the patients with VGAM treated with endovascular embolization had a normal neurological development, about 15% were moderately retarded and only about 10% of the surviving patients show neurological deficits with a severe disability. 23 children died [6].

In another study of Jones et al. over the period of the years from 1987 to 2001, 7 of 13 patients (2 of 8 patients presented as neonates with heart failure and the other 5 patients presented outside neonatal period) achieved a normal or near-normal outcome after endovascular embolization [19].

According to the report by Fullerton et al., 14 of 23 children who had undergone endovascular treatment of the VGAM between the years 1983 and 2002 developed well neurologically [20].

Also Ellis et al. recently reported excellent long-term cognitive and functional outcomes after endovascular occlusions of VGAM [21].

Most patients with VGAM have neurological symptoms at the time of diagnosis making the need for treatment obvious. In our case the diagnosis was a result of ultrasonographic screening in an asymptomatic newborn. The timing of first embolization with 6 months of this asymptomatic infant was chosen according to an evidence-based score. The arteriovenous shunt could be closed before onset of symptoms and without complications.

Patients with VGAM presenting symptoms during the neonatal period have a worse prognosis regarding the success of endovascular embolization [10, 19]. However, clinical signs and symptoms are diverse and may differ individually [4].

## Figures and Tables

**Table 1:** *Bicêtre*-score for evaluation of the therapeutically management of neonates with VGAM (Lasjaunias PL et al. (2006) [6]).

Points	Cardiac function	Cerebral function	Respiratory function	Hepatic function	Renal function
5	Normal	Normal	Normal	—	—
4	Overload, no medical treatment	Subclinical, isolated EEG abnormalities	Tachypnoea, finishes bottle	—	—
3	Failure, stable with medical treatment	Nonconvulsive intermittent neurologic signs	Tachypnoea, does not finish bottle	No hepatomegaly, normal hepatic function	Normal
2	Failure, not stable with medical treatment	Isolated convulsion	Assisted ventilation, normal saturation FiO_2_ <0.25	Hepatomegaly, normal hepatic function	Transient anuria
1	Ventilation necessary	Seizures	Assisted ventilation, normal saturation FiO_2_ >0.25	Moderate or transient hepatic insufficiency	Unstable diuresis with treatment
0	Resistant to medical therapy	Permanent neurological signs	Assisted ventilation, desaturation	Abnormal coagulation, elevated enzymes	Anuria

Score <8: no intervention, 8–12: immediate/emergency embolization, >12: delayed treatment over the age of 5 months.

**Table 2:** State of development of the patient after a standardized developmental test for the first year of life (so called *“Münchener Funktioneller Entwicklungstest” (MFED1)*). Examination at the time of 5th and 11th month of life. Data of tested criteria for the stage of development in corresponding age in month.

Criteria for stage of development (data in month)	First examination with 4 months + 18 days of life	Second examination with 11 months + 9 days of life
Crawling	4	9
Sitting	6	12
Running	5	11
Perception	5	12
Taking	6	12
Speech comprehension	—	12
Social behavior	6	12

Current development state (data in month)	5–5.5	11.5
